# DeepInsight: A methodology to transform a non-image data to an image for convolution neural network architecture

**DOI:** 10.1038/s41598-019-47765-6

**Published:** 2019-08-06

**Authors:** Alok Sharma, Edwin Vans, Daichi Shigemizu, Keith A. Boroevich, Tatsuhiko Tsunoda

**Affiliations:** 1 Laboratory for Medical Science Mathematics, RIKEN Center for Integrative Medical Sciences, Yokohama, Japan; 2 Institute for Integrated and Intelligent Systems, Griffith University, Brisbane, Australia; 3 School of Engineering & Physics, University of the South Pacific, Suva, Fiji; 4 CREST, JST, Tokyo, Japan; 5 Division of Genomic Medicine, Medical Genome Center, National Center for Geriatrics and Gerontology, Obu, Aichi Japan; 6 Department of Medical Science Mathematics, Medical Research Institute, Tokyo Medical and Dental University, Tokyo, Japan; 7 Laboratory for Medical Science Mathematics, Department of Biological Sciences, Graduate School of Science, The University of Tokyo, Tokyo, Japan; 8 School of Electrical and Electronics Engineering, Fiji National University, Suva, Fiji

**Keywords:** Bioinformatics, Computational models, Computational science

## Abstract

It is critical, but difficult, to catch the small variation in genomic or other kinds of data that differentiates phenotypes or categories. A plethora of data is available, but the information from its genes or elements is spread over arbitrarily, making it challenging to extract relevant details for identification. However, an arrangement of similar genes into clusters makes these differences more accessible and allows for robust identification of hidden mechanisms (e.g. pathways) than dealing with elements individually. Here we propose, DeepInsight, which converts non-image samples into a well-organized image-form. Thereby, the power of convolution neural network (CNN), including GPU utilization, can be realized for non-image samples. Furthermore, DeepInsight enables feature extraction through the application of CNN for non-image samples to seize imperative information and shown promising results. To our knowledge, this is the first work to apply CNN simultaneously on different kinds of non-image datasets: RNA-seq, vowels, text, and artificial.

## Introduction

In the post-genomic era, though an abundance of data is accessible, the information is indiscriminately spread over across high dimensional data space, making it challenging to differentiate phenotypes. The same problem of associating relevant features towards a class label lies for other kinds of data (e.g. vowels, text). It becomes critical to arrange elements in an appropriate manner which can enable extraction of relevant features for analyses. Accordingly, the arrangement of information turns to be an important phase via sorting and positioning of the elements in the right order for the subsequent step. We refer this phase as the element arrangement step. The identification or classification of phenotypes or class labels can conceivably be improved following the three steps: element arrangement, feature extraction and developing a suitable classifier.

The conventional machine learning (ML) techniques for classification or detection problem, requires a sample in the form of a feature vector (i.e., a column vector of size *p* × 1). This feature vector obtained from a feature extraction technique is processed to be categorized into one of the defined groups. The features in this vector form are generally considered mutually independent (particularly in the order of appearance) by ML techniques. Consequently, changing the order of features bears no direct impact in classification or phenotype detection, which makes the element arrangement step redundant for many state-of-the-art ML classifiers like random forest^1,2^ and decision trees^3^. However, the reliability of ML techniques is dependent on the feature extraction technique.

On the other hand, convolution neural network (CNN) architecture from deep neural networks accepts a sample as an image (i.e. a matrix of size *m* × *n*) and performs feature extraction and classification via hidden layers (such as convolutional layers, RELU layer, max-pooling layers). It does not require additional feature extraction techniques as it automatically derives features from the raw elements. The second advantage is that it finds higher-order statistics of image and nonlinear correlations. Third, convolutions neurons process data for its receptive fields or restricted subarea, relaxing the need to have a very high number of neurons for large input sizes and therefore enables the network to be much deeper with fewer parameters^4^. Another distinguishing attribute of CNN is weight sharing; i.e., many receptive fields share the same weights and biases (or filter), enabling a reduction in the memory footprint as compared to conventional neural networks. The CNN architecture allows to deal with images effectively and becoming a promise in accuracy for industrial applications (such as driverless cars). The image consists of spatially coherent pixels in a local region; i.e., the pixels close to each other share similar information. Subsequently, the positioning of respective pixels can adversely affect the feature extraction and classification performance of CNN architecture if arbitrarily arranged. Therefore, the order of neighboring pixels in an image utilized by CNN are no longer independent as they were in ML techniques. Additional information is captured at a time of process when CNNs employ a collection of neighboring pixels as opposed to individual use of features by ML techniques. The credit of success also goes to the hardware advancements such as GPUs, which allow very complex models to be trained in a much faster and affordable manner. Also, the development of new deep learning architectures and libraries enable models to be built and learned rapidly. Fortunately, for CNNs, captured images generally are a depiction of physical objects and don’t require rearrangement of pixels as camera lenses place the corresponding shades of objects rightly on to the pixels.

A lot of data such as genomic, transcriptomic, methylation, mutation, text, spoken words, financial and banking are in non-image form and ML techniques are dominantly used in these fields. Moreover, CNN can’t be used because it requires an image as an input. However, if we can transform non-image data to a well-organized image form, then CNN can be used for higher classification performance. For this, we need to develop a method that can perform element arrangement effectively. To improve the detection rate, we integrated all the three steps (element arrangement, feature extraction and classification) in the proposed DeepInsight method. DeepInsight, constructs an image by placing similar elements or features together and dissimilar ones further apart, enabling the collective use of neighboring elements. This collective approach of element arrangement can be useful in uncovering hidden mechanisms (e.g. pathways) or understanding relationship between a set of features (e.g. for texts, vowels). Therefore, conversion to an image by inserting alike features (or raw elements) as clusters is more meaningful and robust than dealing with individual features (ignoring neighborhood information) as important information (from weak elements) can be integrated. This has a potential to explore the relative importance of features towards a target or outcome. Element arrangement is a key to unlock crucial information. It is pertinent to ponder upon strategies which may retrieve more information from a given dataset. Furthermore, DeepInsight, allows feature extraction and classification via the utilization of CNN. This will increase the versatility of CNN by opening it to non-image cases and thus provide a generalized outcome of CNN. We show in this paper that DeepInsight has usefulness for various kinds of data like gene-expression, vowels, texts and artificial.

Different versions of CNNs have been proposed to deal with images effectively^5–16^. For example, He *et al*.^8^, proposed a residual networks architecture to make it easier to train very deep networks. They used 152 layers deep residual on the ImageNet dataset. Singh *et al*.^17^ developed CNN based technique to classify gene expression using histone modification data as input. Liu *et al*.^18^ used tumor gene expression samples as a column vector and employed 1-dimensional CNN to perform classification. They did not convert samples to images. Zeng *et al*.^19^ applied CNN to extract features from *in situ* hybridization gene expression patterns. The input samples were natural images. Gao *et al*.^20^ uses DNA sequences and convert into 4-dimensional binary codes. These binary codes are arranged according to the DNA sequence and then applied to CNN to predict polyadenylation sites. Xu *et al*.^21^ applied CNN on text hashing where texts are converted into binary coding and then fed to 1-dimensional convolution; i.e., these features are no longer treated as images in convolutional layers. Zhang *et al*.^22^ perceived text as a raw signal and applied 1-dimensional CNN for classification. Lyu and Haque^23^ have recently applied CNN for RNA-seq data by first performing gene selection followed by constructing an image based on chromosome location. This method is perhaps the first one of converting gene expression into image samples and applying CNN for classification. Since this method requires chromosome location information, it is not possible to use it for other kinds of datasets. Most of the methods discussed above are either applied images as input to CNN or used 1-dimensional CNN. Therefore, minimal literature is available to ubiquitously convert non-image samples to images for the applications of CNN.

## Results

### Experimental setup

We employed four different kinds of datasets to test the DeepInsight method, and we also compared the obtained results of it with the state-of-the-art classifiers. There are 1 gene-expression dataset, 1 text dataset, 1 vowels dataset and 2 artificial datasets. The prime objective is to show that a non-image data can be processed by utilizing the CNN architecture through the implementation of the DeepInsight method.

The datasets considered for this work are first subdivided into training, validation and test sets using 80:10:10 proportion, respectively. Fitting of a model is carried out on the training set, and its fitness is evaluated on the validation set. The hyperparameters are selected for which the validation error is minimum. The test set has never been employed in the training or model fitting step. The classification accuracy is computed on the test set to deliver an unbiased assessment of a final model, where classification accuracy is defined as the percentage of the number of samples correctly classified from the test set.

The description of these datasets is as follows. The first is an RNA-seq or gene expression dataset which is a public dataset from TCGA (https://cancergenome.nih.gov) containing 6216 samples, and each sample is of 60483 genes or dimensions. This is a 10-class dataset, representing ten types of cancer. The second is a speech dataset from the TIMIT corpus^24,25^. Here a set of 10 distinct monophthong vowels are extracted, then each vowel is subdivided into three segments, and each segment is used to generate mel-frequency cepstral coefficients with energy-delta-acceleration (MFCC_EDA) feature vectors. A total of 12579 samples with 39 dimensions are obtained. The third dataset is Relathe (text)^26^ which is derived from newsgroup documents and partitioned evenly across different newsgroups. It contains 1427 samples and 4322 dimensions. It is a two class problem. The next two are artificial datasets. One is Madelon^27^ which has 2600 samples and 500 dimensions. This is a two-class classification problem with continuous input variables. It is multivariate and highly non-linear. The other is ringnorm-DELVE, which is an implementation of Leo Breiman’s ringnorm example^28^. It is 20 dimensional, 2 class classification with 7400 samples. Each class is drawn from a multivariate normal distribution, where class 1 has zero mean and covariance four times the identity, and class 2 has a mean *a* = 2/sqrt(20) with unit covariance. These datasets are summarized in Table 1.

**Table 1:** Summary of Datasets.

Datasets	#samples	#features	#classes
RNA-seq	6216	60483	10
Vowels	12579	39	10
Relathe	1427	4322	2
Madelon	2600	500	2
Ringnorm-DELVE	7400	20	2

### Comparison and classification performance

The existing state-of-the-art classifiers such as random forest, decision tree, and ada-boost were employed for comparison purposes. The hyperparameters of competing methods were optimized using grid search optimization. As discussed in Section 4.3 and Supplement File 1, the DeepInsight method employs two types of normalizations (norm-1 and norm-2), and the validation error is evaluated on both these norms. The norm which gives the lowest validation error is used for further processing. The pixel frame size is fixed at 120 × 120. However, for RNA-seq dataset, the analysis was done on 200 × 200 pixel size since the number of elements or features is very large (60483) resulting in lossy compression (as discussed in Supplement File 2) compared to other datasets studied in this work. The validation errors for both the norms are depicted in Supplement File 3 for all the datasets after executing DeepInsight. The best fit model on the validation set is used to evaluate performance on a separate test set.

The purpose of this comparison is to show that DeepInsight can also produce a competitive performance on different kinds of datasets. The performance regarding classification accuracy is depicted in Table 2 (see Supplementary File 4 for a brief discussion on codes).

**Table 2:** Classification accuracy on different kinds of datasets using various models.

Datasets	Decision Tree	Ada-Boost	Random Forest	DeepInsight
RNA-seq	85%	84%	96%	**99%**
Vowels	75%	45%	90%	**97%**
Text	87%	85%	90%	**92%**
Artificial (Madelon)	65%	60%	62%	**88%**
Artificial (Ringnorm-DELVE)	90%	93%	94%	**98%**
*Average*	*80%*	*73%*	*86%*	*95%*

DeepInsight produces 99% classification accuracy on a test set of RNA-seq data which is 3% more than the state-of-the-art random forest method. For vowels dataset, DeepInsight scored 97% classification accuracy compared to 90% by random forest. This improvement is around 7% better than the best performing existing method compared in this study. Next, on text data, DeepInsight obtained 92% accuracy compared to 90% achieved by random forest method. The same trend can be found in for artificial datasets: Madelon and ringnorm. On Madelon, DeepInsight obtained 88%, and on ringnorm, it achieved 98%. The improvement is 23% and 4%, respectively, compared to the second best technique. The average classification accuracy over all the five datasets is also computed. Ada-boost obtained 73% average classification accuracy while decision tree scored 80% which is better than the ada-boost method. Random forest achieved 86% which is the best out of the existing techniques studied, whereas, DeepInsight scored a promising average classification accuracy of 95% which is significantly better than the performance of the second best method.

## Discussion

As anticipated, the proposed DeepInsight method produced very promising results. The obtained results enable us to use CNN architecture for various kinds of non-image datasets. This increases the possibility of utilizing deep learning networks. One can envisage the immense possibility of applying this algorithm to a wide variety of applications.

In this work, we were able to integrate many properties of CNN for non-image samples through the inception of DeepInsight method. A non-image sample, in the form of vectors were transformed into meaningful images for the processing of CNN. This strategy does not solve all problems related to genomic data, however, it is a step forward in integrating the merits of CNN. Deep neural network architectures, encompass many advantages: feature extraction, dimension reduction, finding hidden structure from sparse and hyper-dimensional data, data augmentation and up-sampling, semi-supervised learning with labeled/non-labeled samples, and optimum action selection with time-series data^29^. Therefore, for a broader context, deep neural network architectures have the potential to offer a solution for genomic analysis for a variety of input samples ranging from DNA sequences to protein sequences (which may be considered as time-series data) to RNA-seq or omics data.

The DeepInsight method increase the versatility of CNN architectures. The characteristics of CNN such as automatic feature extraction, reducing the need of neurons and consequently enabling to train a model much deeper, weight sharing capability to mitigate memory requirement, utilization of neighborhood information (i.e., processing subarea of pixel frame at a time), and, GPU utilization make CNN a potent tool for classification and analysis. These attributes of CNN are utilized for non-image cases by the proposed technique. Further, we have shown the effectiveness of DeepInsight on several kinds of datasets and obtained very promising results. For RNA-seq data the maximum classification accuracy achieved by DeepInsight was 99%. For vowels, text, Madelon and ringnorm the accuracies were 97%, 92%, 88% and 98%, respectively.

Further extensions of the current version of the algorithm can be considered. The present technique employs gray-scale or single layer (i.e., 2D matrix) for classification. This can be extended to incorporate multiple layers and therefore can be applied to solve problems related to multi-omics data (e.g., gene-expression, methylation, mutation) as well. Moreover, different kinds of data (e.g., clinical and non-clinical) can be normalized into a single layer (if multi-layer is prohibited due to computing resources) for analysis and classification. This technique can be useful for a number of applications where data is not in image form.

## Methods

### DeepInsight method

**Figure 1:**
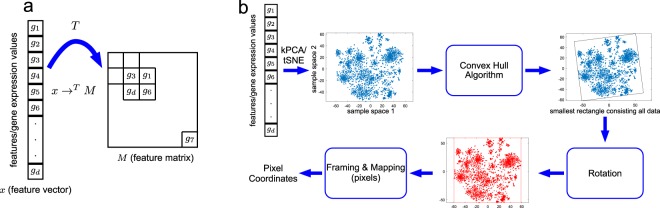
DeepInsight pipeline. (**a**) An illustration of transformation from feature vector to feature matrix. (**b**) An illustration of the DeepInsight methodology to transform a feature vector to image pixels.

The concept of DeepInsight is to first transform a non-image sample to an image form and then supply it to the CNN architecture for the prediction or classification purpose. A simple illustration is given in Fig. 1a, where a feature vector *x* consisting of gene expression values is transformed to a feature matrix *M* by a transformation *T*. The location of features in the Cartesian coordinates depends on the similarity of features. For example, features g1, g3, g6 and gd are closer to each other in Fig. 1a. Once the locations of each feature are determined in a feature matrix, then the expression values or feature values are mapped. This will generate a unique image for each sample (or feature vector). *N* samples of *d* features will provide *N* samples of *m* × *n* feature matrices. This 2D matrix form will have all the *d* features. Thereafter, this set of *N* feature matrices are processed to the CNN architecture for learning the model and providing prediction.

If the data dimensionality is extremely large and difficult to handle due to hardware limitations, then the dimensionality reduction technique (DRT) may be considered before applying DeepInsight. The DRT can be either in the form of feature selection or feature extraction depending upon the nature of the problem. The application of DRT will provide a small feature set which will help in faster processing, however, can risk classification performance. On the other hand, if noisy or redundant features are removed then it could help to get higher processing speed as well as better accuracy. Since the application of DRT is case dependent, we have described DeepInsight without applying DRT.

### DeepInsight pipeline

In this section, we briefly discuss the transformation of a non-image sample to its image form. A general overview of this transformation is depicted in Fig. 1b. The training set is used to find the location of features. If the training set consisting of *n* samples is defined as *χ* = {*x*_1_, *x*_2_, …, *x*_*n*_} where a feature vector has *d* features or $x\in {{\mathbb{R}}}^{d}$, then we can also define a gene or feature set *G* = {*g*_1_, *g*_2_, …, *g*_*d*_} where $g\in {{\mathbb{R}}}^{n}$; i.e., a feature *g*_*j*_ has *n* training samples. Basically, *G* can be obtained by transposing *χ*. We used this feature set *G* and applied similarity measuring technique or dimensionality reduction technique like t-SNE^30^ or kernel principal component analysis (kPCA) to obtain a 2D plane (please see details about t-SNE and kernel PCA in Supplement File 5. These are non-linear dimensionality reduction techniques. A number of linear dimensionality reduction techniques also exist but not implemented in this work^31–34^). The points in this Cartesian plane are the features or genes. These points only define the location of features, not the feature itself or expression values. Once the location of features is defined, the convex hull algorithm is used to find the smallest rectangle containing all the points. Since the image should be framed in a horizontal or vertical form for the CNN architecture, a rotation is performed. Thereafter, the Cartesian coordinates are converted to pixels. The conversion from Cartesian coordinates to pixel frames is done by averaging some features as the image size has a pixel limitation. The pixel frame will, therefore, consist of the positions of features (or genes) for a sample *x*_*j*_ (for *j* = 1, 2, …, *n*). Once the location is determined, the next step is to map the feature (or gene expression) values to these pixel locations. If more than one feature acquired the same location in the pixel frame, then, during mapping of the features, the respective features will be averaged and placed in the same location. Therefore, if the resolution of image or grid size is very small (compared to the number of features given), then many features overlap with each other and image representation may not be very accurate. An appropriate resolution should be selected given the hardware capacity and the number of features required to process. Alternatively, dimensionality reduction may be applied a priori. The details about the procedure is given in Supplementary File 6.

### Feature normalization

The single layer of the image has 256 shades which are normalized in the range of [0, 1]. Therefore, feature values are to be normalized before applying the image transformation. In this work, we performed two types of normalizations: (1) each feature is assumed independent and therefore normalized by its minimum and maximum, and (2) the topology of mutual features are retained up to some extent by normalizing it with the one maximum value from the entire training set. These normalizations are explained in detail in the Supplementary File 1. DeepInsight evaluates validation set performance on both the types of normalizations and accepts the one with the lowest validation error.

### CNN architecture

**Figure 2:**
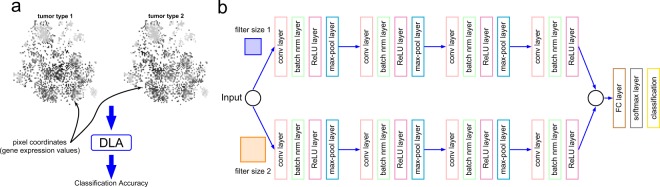
DeepInsight network: an illustration. (**a**) Illustration of two types of tumors using the image transformation methodology of the DeepInsight method. The difference between the two types can be visualized at various points. These image samples are further processed to deep learning architecture (DLA); i.e., parallel CNN as depicted in part b of the same figure. (**b**) Parallel CNN architecture used in DeepInsight. This architecture consists of two parallel CNN architectures where each consists of four convolutional layers. Parameters are tuned using Bayesian Optimization technique.

In this section, we describe the CNN architecture of the DeepInsight method. Once a feature vector is transformed into an image, it can then be further processed to the CNN architecture (an illustration of two types of cancer samples in image form is shown in Fig. 2a).

We developed a parallel CNN architecture so that different filter-sizes can be effectively used to train the model. Our CNN architecture is shown in Fig. 2b. In this architecture, we have four layers in parallel where each layer consists of a 2D convolution layer, a batch normalization layer, a ReLU activation layer, and a max pooling layer. Batch normalization is used to prevent overfitting during training, and the max pooling layer is used to down-sample the image size in each layer. The outputs of the fourth convolution layer (in the parallel architecture) are combined and fed to a fully connected layer. Finally, a SoftMax layer is used to give the output as class labels.

The CNN architecture of DeepInsight has various hyperparameters such as convolution layers, filter sizes, learning rate and so on. We tuned these hyperparameters by applying Bayesian optimization technique for all the trials. We obtained a set of hyperparameters that gave the best performance on the validation set. The parameter details and validation error during the training phase are discussed in Supplementary File 2 and Supplement File 3.

Once the CNN model is trained using the optimal hyperparameters, then any novel sample can be identified into one of the categories or classes.

**Figure 3:**
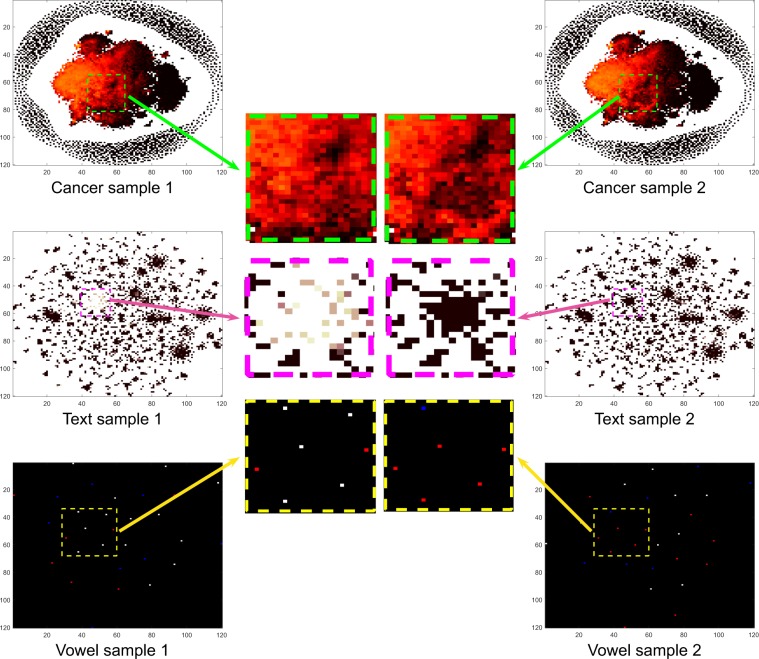
Revealing patterns by DeepInsight. An illustration showing the different patterns achieved by DeepInsight on gene-expression (different kind of cancers), text (two types of text) and vowels (two types of vowels). Each plot shows a transformed sample, the difference between samples can now be noticed straightforwardly.

For an illustration, two samples are derived from distinct types of cancers, texts and vowels to observe the difference between samples. The transformed samples by DeepInsight method are shown in Fig. 3. This method provides interesting localities by performing element arrangement, then feature dissimilarity is further captured by feature extraction and classification through the application of CNN. Moreover, these samples can now be visualized, and their relative difference in particular regions might lead to different class labels (or phenotypes).

## Supplementary information

Normalizations used for DeepInsight, Parameters of DeepInsight, Results, Codes description, Non-linear dimensionality reduction techniques, Description of DeepInsight Pipeline

## Data Availability

RNA-seq data is available from TCGA (https://cancergenome.nih.gov). Vowels data can be extracted from TIMIT Acoustic-Phonetic Continuous Speech Corpus (https://catalog.ldc.upenn.edu/LDC93S1). Text data is available from http://featureselection.asu.edu/datasets.php. Madelon dataset is available from UCI repository http://archive.ics.uci.edu/ml/datasets/madelon, and ringnorm dataset is available from University of Toronto at https://www.cs.toronto.edu/~delve/data/ringnorm/desc.html.

## Code Availability

All the source code, a sample dataset and documentaries are available at http://www.riken.jp/en/research/labs/ims/med_sci_math/ or http://www.alok-ai-lab.com.
