# Supplementary material for: DeepInsight: A methodology to transform a non-image data to an image for convolution neural network architecture
Source: Sci Rep. 2019 Aug 6;9:11399. doi: 10.1038/s41598-019-47765-6 (PMC6684600; doi:10.1038/s41598-019-47765-6)
Supplement: Supplementary file 1 — Normalizations used for DeepInsight, Parameters of DeepInsight, Results, Codes description, Non-linear dimensionality reduction techniques, Description of DeepInsight Pipeline [file 41598_2019_47765_MOESM1_ESM.pdf]

DeepInsight: A methodology to transform a non-image data to an image for convolution neural network architecture

Alok Sharma[1,2,3,*], Edwin Vans[3], Daichi Shigemizu[1,4,5,6], Keith A Boroevich[1], Tatsuhiko Tsunoda[1,4,6,7*]

[1]Laboratory for Medical Science Mathematics, RIKEN Center for Integrative Medical Sciences, Japan.
[2]Institute for Integrated and Intelligent Systems, Griffith University, Australia.
[3]School of Engineering & Physics, University of the South Pacific, Fiji
[4]CREST, JST, Tokyo, Japan
[5]Division of Genomic Medicine, Medical Genome Center, National Center for Geriatrics and Gerontology, Obu, Aichi, Japan.
[6]Department of Medical Science Mathematics, Medical Research Institute, Tokyo Medical and Dental University, Tokyo, Japan.
[7]Laboratory for Medical Science Mathematics, Department of Biological Sciences, Graduate School of Science, The University of Tokyo, Tokyo, Japan.

*corresponding authors: alok.fj@gmail.com, tsunoda.mesm@mri.tmd.ac.jp .

Supplementary File 1

Normalizations used for DeepInsight

Here we describe two types of normalization used in this work: norm-1 and norm-2. For norm-1, each feature is normalized by its minimum and maximum. This will bring a feature between 0 and 1. This type of normalization will assume that features are mutually independent as a feature is normalized by its extrema values. The minimum and maximum values for norm-1 can be computed in the following manner.

$$\mathrm{Max}_j = \max_{samples} X_{tr}(j,:)$$
$$\mathrm{Min}_j = \min_{samples} X_{tr}(j,:)$$

Where X_{tr} is the training set and $(j,:)$ refers to all the samples of the jth feature or attribute. Therefore, Max_j and Min_j are the maximum and minimum of the jth attribute. These extrema values are used to normalize training, validation and test sets as

$$X_{tr}(j,:) = \frac{X_{tr}(j,:) - \mathrm{Min}_j}{\mathrm{Max}_j - \mathrm{Min}_j}$$
$$X_{val}(j,:) = \frac{X_{val}(j,:) - \mathrm{Min}_j}{\mathrm{Max}_j - \mathrm{Min}_j}$$
$$X_{test}(j,:) = \frac{X_{test}(j,:) - \mathrm{Min}_j}{\mathrm{Max}_j - \mathrm{Min}_j}$$

Where $j = 1,2, \ldots, d$, and d is the dimension of the samples in the dataset. If after normalization any feature value of the validation set or test set is less than 0 or greater than 1, then such feature values are clamped between 0 and 1 to maintain the consistency.

In the norm-2 normalization will try to keep the topology of the features up to some extent. In this method, the minimum value is adjusted for each feature or attribute, and then a global maximum is used in the

logarithmic scale to place the feature values between 0 and 1. The norm-2 is conducted in the following manner:

$$\text{Min}_j = \min_{samples} X_{tr}(j,:)$$
$$X_{tr}(j,:) \leftarrow \log(X_{tr}(j,:) + |\text{Min}_j| + 1)$$
$$\text{Max} = \max(X_{tr})$$
$$X_{tr}(j,:) \leftarrow \frac{X_{tr}(j,:)}{\text{Max}}$$

The validation and test sets are adjusted using the training extrema values for normalization. In case, after adjusting by the minimum values (Min_j), any element of validation or test set is less than 0 then it is clamped at 0. Similarly, if after normalizing by the maximum value (Max) any feature from the validation and test sets are above 1 then it is clamped to 1.

Supplementary File 2

Parameters for DeepInsight

In this supplement, we describe parameters used for DeepInsight method.

<u>CNN parameter</u>

Four convolution layers are implemented in a parallel configuration. The Bayes optimization technique is used to find the best parameters from a range of values used. The filter size or window size for each parallel layer is different. The parameters, like the number of filters, momentum, and l2-regularization, are same. The maximum objective evaluation and maximum epochs are set to 100. The parameters are summarized in Table S2.1

Table S2.1: Training parameter options for DeepInsight method

Variables	*Values/range*
FilterSize-1	[2 8] ~ [2 10]
FilterSize-2	[4 20] ~ [4 30]
InitialNumFilters	[2 16] ~ [4 16]
Momentum	[0.8 0.95]
L2regularization	[1e-10 1e-2]
MaxObjectiveEvaluation	100
MaxTime	8x60x60 ~ 24x60x60
ExecutionEnvironment	Multiple-gpu
LearningRateSchedule	Piecewise
LearningRateDropPeriod	35
LearningRateDropFactpr	0.1
InitialLearnRate	[1e-3 1e-1] ~[1e-5 1e-1]
MiniBatchSize	128
PoolSize	[2 2]
Stride	[2 2]

The range of values are applied during the training session and best the values were selected which gave the least validation error.

<u>Dimensionality reduction technique</u>

We utilized t-SNE and kernel PCA for finding locations of features. In case of t-SNE, if the number of features is less than 5000 then the exact algorithm is used otherwise Burneshut algorithm is applied (to speed up processing). The default distance in t-SNE is 'cosine'. For kernel PCA two eigenvectors

corresponding to the leading eigenvalues are used to do transformation. The kernel type used was 'Gaussian'.

Feature mapping

Once the feature locations are defined using the training set, the next step is to map feature values to these locations. If two or more than two features occupy the same location then their averaged values are used; i.e., if locations of g_i, g_j and g_k are the same (a, b) then $(g_i + g_j + g_k)/3$ will be mapped on this location. This will allow lossy compression of features. The validation and test sets use the feature locations obtained using the training set. For the empty pixels; i.e., pixels that do not contain any features are referred as Base, and its value is fixed as 1.

Pixel frame

The pixel size can be arranged automatically or can be fixed. The auto-mode determines the size $A \times B$ by utilizing the distance of two nearest feature location (referred as d_{min} in equations S6.3 and S6.4 of Supplement File 6). However, this will enlarge the pixel size and therefore it is limited by maximum predefined size of either A or B. If maximum size is M the the pixel size is adjusted accordingly. In this work, we used maximum pixel size as 120×120 and 200×200.

Supplementary File 3

Results

A dataset is first partitioned into three segments, namely train, validation and test sets. The proportion of train, validation and test is roughly 80:10:10. All the results are on test sets.

For DeepInsight method, we optimized the parameters using train and validation sets. The parameters selected are those for which the validation error is minimum. DeepInsight method employs norm-1 and norm-2 normalization (as described in the manuscript and Supplement File 1) and the validation error is evaluated on both these norms, and the norm which provided the lowest validation error is used. The validation errors for both the norms are depicted in Table S3.1 .

Table S3.1: Validation error on all the datasets using pixel size 120×120.

Datasets	Norm-1	Norm-2
RNA-seq	0.0179	0.0161
Vowels	0.0292	0.0425
Relathe	0.1875	0.1484
Madelon	0.1667	0.2650
Ringnorm-DELVA	0.0015	0.0015

The validation error for RNA-seq dataset when pixel size is 200×200 was also obtained. The values for norm-1 is 0.0233 and for norm-2 it is 0.0179; i.e., norm-2 is selected in this case due to lower validation error. The test accuracy obtained was 99%.

For pixel size 120×120 the test accuracies obtained are depicted in Table S3.2

Table S3.2: Accuracy on test set when pixel size 120×120 is used.

Datasets	Accuracy
RNA-seq	98%
Vowels	97%
Relathe	92%
Madelon	88%
Ringnorm-DELVA	98%

Supplementary File 4

Codes description

This package is written in Matlab. It has two main components: transforming into pixels and processing via convolution neural networks (CNNs). A summary of the code and how to use it is discussed herein. As an example dataset, ringnorm-DELVE is provided with the package.

1) Dataset *struct*

The dataset (dset) should be in the following *struct* format

Xtrain	[d x n] (where d is the number of features and n is the number of training samples)
Xtest	[d x m] (where m is the number of test samples)
num_tr	[p1, q1, r1 …] (number of samples in each class for training dataset)
num_tst	[p2, q2, r2, …] (number of samples in each class for test dataset)
class	number of classes or categories
dim	number of features or attributes d
Set	name of the dataset

The training samples are arranged in the following manner. All samples belong to one class are kept first, then the samples of 2nd class is positioned and so on. The same was done for the test set.

2) Main.m is the main file. It requires the following parameters to set

Parm.fid	Results.txt file will register the output
Parm.Method	Select dimensionality reduction methods from 'tSNE', 'kPCA', 'PCA'
Parm.Max_Px_Size	The maximum pixel size max([A,B])
Parm.MPS_Fix	If this value is 1 then pixel size will be PxP otherwise MxN or NxM where M (if $M > N$) is Max_Px_Size and N is determined by eq S6.3/S6.4 (see Supplement File 6).
Parm.ValidRatio	Define ratio of validation over train.
Parm.Seed	Random seed to split training and validation sets.

3) Training and test of DeepInsight model

DeepInsight_train is used for training the model and DeepInsight_test is to evaluate the accuracy

Usage:

Model = DeepInsight_train(dset, Parm)	Only training samples and Parm are required
Accuracy = DeepInsight_test(dset,model)	Only test samples and model are required

4) Transformation to pixel image form

Cart2Pixel and ConvPixel are used to convert Cartesian coordinates to pixel frames.

5) Bayesian optimization parameter for convolution neural network

Two parallel layers are used. See Table S2.1 (Supplement File 2) for details about range of parameters explored. It is required to set the parameters as desired. Layer connections can be modified by changing functions in makeObjFcn2.m file.

Network models are stored in DeepResults folder during the run time. The dataset is placed in Data folder.

Supplementary File 5

Non-linear dimensionality reduction techniques

In this supplement, we describe two non-linear dimensionality reduction techniques employed in the DeepInsight method. These techniques are t-distributed stochastic neighbor embedding (t-SNE) (Maaten and Hinton, 2008) and kernel principal component analysis (PCA) (Schölkopf et al., 1998).

t-SNE

The t-SNE technique visualizes high-dimensional data on a two or three dimensional plane for clustering samples.

The mapping from higher dimensional space to lower dimensional space happens in a non-linear fashion. The samples with similarity, map close to each other, and with dissimilarities mapped apart. This technique is a variant of stochastic neighbor embedding (Hinton and Roweis, 2002) and easier to optimize, leading to better visualizations.

Many linear dimensionality reduction techniques map data to a 2D plane (DeepInsight does not require 3D transformation by t-SNE). However, mapped samples are highly convoluted, and it becomes very challenging for clustering algorithms to find a reasonable level of groupings. On the other hand, this technique has the potential to map very high dimensional data to a 2D plane while trying to keep the topology, or in other words, with minimum error. This enables understanding of complex data structures in a lower dimensional space. However, the processing time of t-SNE can be prolonged. For faster processing, the Barneshut algorithm is used to approximate joint distributions instead of the exact computation.

The t-SNE technique has two main steps. In the first step, it constructs a probability distribution over pairs of samples such that similar samples have higher probability and dissimilar samples have lower probability. In the second step, it finds the probability distribution in a 2D plane. Then it minimizes the Kullback-Leibler divergence between the two distributions belonging to lower- and higher-dimensional spaces using a gradient descent method.

t-SNE uses Euclidean distance (however, in DeepInsight, cosine distance was used) to compute probabilities. The conditional probability, $p_{j|i}$, used in t-SNE, is a measure of the probability that a sample x_i will pick x_j as its neighbor under Gaussian distribution. It can be defined as

$$p_{j|i} = \frac{\exp(-\left\|x_i - x_j\right\|^2 / 2\sigma_i^2)}{\sum_{k \neq i} \exp(-\left\|x_i - x_k\right\|^2 / 2\sigma_i^2)} \qquad \text{(S5.1)}$$

where $x \in \mathbb{R}^d$ and σ_i is the variance of the Gausssian that is centered at sample x_i. Since t-SNE is only interested in pairwise probability, $p_{i|i}$, has been set to 0.

Similarly, the conditional probability in 2D-plane, $q_{j|i}$, for mapped samples y_i and y_j (where $y \in \mathbb{R}^2$), can be given as

$$q_{j|i} = \frac{\exp(-\left\|y_i - y_j\right\|^2 / 2\sigma_{yi}^2)}{\sum_{k \neq i} \exp(-\left\|y_i - y_k\right\|^2 / 2\sigma_{yi}^2)} \qquad \text{(S5.2)}$$

The variance is set to $1/\sqrt{2}$, therefore, $2\sigma_{yi}^2 = 1$.

If the conditional probabilities, $p_{j|i}$ and $q_{j|i}$, are equal, then it means sample points, y_i and y_j, correctly model the similarity between higher dimensional samples, x_i and x_j. Therefore, the aim is to model $q_{j|i}$ as close as of $p_{j|i}$. This is done by minimizing Kullback-Leibler divergence with respect to y_i, using a gradient descent method as

$$C = \sum_i KL(P_i||Q_i) = \sum_i \sum_j p_{j|i} \log \frac{p_{j|i}}{q_{j|i}} \quad \text{(S5.3)}$$

where C is the cost function, KL is the Kullback-Leibler divergence function, P_i is the conditional probability distribution over all samples given x_i, and Q_i is the conditional probability distribution over all mapped samples given y_i.

As a consequence of this optimization, mapped samples in the lower-dimensional space can be found which similitudes samples between the higher-dimensional space.

Kernel PCA

Kernel PCA is beneficial for visualization, novelty detection and image de-noising. It is an extension of the PCA technique for dimensionality reduction by incorporating kernel functions. These kernel functions help to compute the principal components in much higher dimensional spaces. However, the transformation to these higher dimensional spaces does not explicitly occur.

In kernel PCA, projection function ϕ is used to transform samples $x \in \mathbb{R}^d$ to a feature space. This feature space could be in infinite dimensional space. However, instead of explicitly computing this feature space, kernel trick is used to obtain samples $y \in \mathbb{R}^h$ (where $h < d$) in a parsimonious data space.

Assuming a projected dataset with N samples $\phi(x_1), \phi(x_2), \ldots, \phi(x_N)$ are centered, and, therefore, its mean is zero. The covariance matrix can be obtained using as

$$\Sigma = \frac{1}{N} \sum_{i=1}^{N} \phi(x_i)\phi(x_i)^T = E[\phi(x)\phi(x)^T] \quad \text{(S5.4)}$$

where $E[\dot{}]$ is an expectation function, and $\phi(x)$ is a sample from this dataset. If the data matrix is denoted by Φ then $\Phi = [\phi(x_1), \phi(x_2), \ldots, \phi(x_N)]$, which will allow equation (S5.4) to write in a matrix form as

$$\Sigma = \frac{1}{N} \Phi\Phi^T \quad \text{(S5.5)}$$

Eigenvalue decomposition (EVD) of covariance matrix Σ will give

$$\Sigma v_i = \lambda_i v_i, \quad \text{for } i = 1,2, \ldots, d \quad \text{(S5.6)}$$

Since v_i can be represented as a linear combination of $\phi(x_1), \phi(x_2), \ldots, \phi(x_N)$, we can write $v_i = \Phi u_i$, where u_i is a N-dimensional column vector. Using this equality and from equation (S5.5), we can rewrite equation (S5.6) as

$$\frac{1}{N} \Phi\Phi^T \Phi u_i = \lambda_i \Phi u_i$$

Multiplying LHS by Φ^T, would give,

$$\frac{1}{N}\Phi^T\Phi\Phi^T\Phi u_i = \lambda_i\Phi^T\Phi u_i \quad \text{(S5.7)}$$

It is easy to eliminate the term $\Phi^T\Phi$ from both the sides in equation (S5.7). Also if we define kernel $K = \Phi^T\Phi$, then

$$\frac{1}{N}Ku_i = \lambda_i u_i \quad \text{(S5.8)}$$

that is, u_i, is the eigenvector of K (an $N\times N$ matrix) corresponding to eigenvalue λ_i. In order for normalization,

$$1 = v_i^T v_i = u_i^T\Phi^T\Phi u_i = u_i^T K u_i = N\lambda_i u_i^T u_i$$

Thereafter, dimensionality reduction can be applied as

$$Y_i = v_i^T\Phi = u_i^T\Phi^T\Phi = u_i^T K \quad \text{(S5.9)}$$

So far, we have assumed that the projected data $\phi(x)$ has a zero mean. But in practice, this is not true. Therefore, projected data after centralizing, denoted $\hat{\phi}(x)$, will give kernel $\hat{K}$ as

$$\hat{K} = K - 1_N K - K 1_N + 1_N K 1_N \quad \text{(S5.10)}$$

where 1_N is an $N\times N$ matrix for which every element takes the value of $1/N$.

There could be a variety of kernel functions. For e.g. linear kernel between two samples, x and x', could be $k(x,x') = x^T x'$, or Gaussian kernel could be $k(x,x') = \exp\left(-\|x - x'\|^2/\rho\right)$.

Reference

L. Maaten and G. Hinton, "Visualizing High-Dimensional Data using t-SNE," *Journal of Machine Learning Research,* vol. 9, pp. 2579-2605, 2008.

B. Schölkopf, A. Smola, K-R. Müller, "Nonlinear Component Analysis as a Kernel Eigenvalue Problem". *Neural Computation*, vol. 10, no. 5, pp. 1299–1319, 1998.

G.E. Hinton and S.T. Roweis, "Stochastic Neighbor Embedding. In Advances in Neural Information Processing Systems", vol. 15, pp. 833–840, Cambridge, MA, USA, 2002. The MIT Press.

Supplementary File 6

Description of DeepInsight Pipeline

An overview of the DeepInsight pipeline is depicted in Figure 1b (main manuscript). Here we describe the details of the pipeline. A dataset was subdivided into 3 parts, namely the training set, validation set and test set. The training set is employed to find the location of attributes or features in a 2D plane. Let a training set consisting of n samples and d attributes be defined as $\chi = \{x_1, x_2, \ldots, x_n\}$. The attributes of χ can be represented as $G = \{g_1, g_2, \ldots g_d\}$ where g is a feature vector with n entries. This set G is processed through t-SNE or kernel PCA to get 2D coordinates $\{(a_1, b_1), (a_2, b_2), \ldots, (a_d, b_d)\}$. The coordinates (a_j, b_j) define the location of g_j, where $j = 1,2, \ldots, d$.

Next, the convex hull algorithm is applied to find the minimum box covering all the points. Since this box is not necessarily in the horizontal or vertical direction (as required by the CNN architect), we perform rotation. For rotation, gradient of two corner coordinates of a rectangle (obtained by the convex hull algorithm) is considered. If the coordinates are defined as (x_{c1}, y_{c1}) and (x_{c2}, y_{c2}) then gradient Gr is defined as (See Figure S6.1)

$$Gr = \frac{y_{c2} - y_{c1}}{x_{c2} - x_{c1}} \qquad \text{(S6.1)}$$

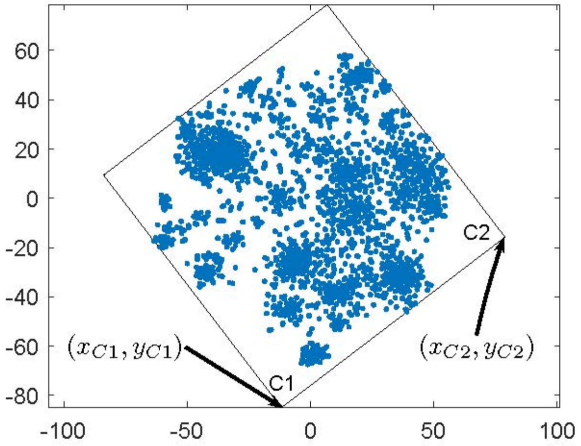

Figure S6.1 Plot showing the smallest rectangle containing all the data points. The two corner points used to compute gradient are shown as C1 and C2

This will enable to compute the rotation angle θ defined as $\theta = \tan^{-1}(Gr)$. This will provide the rotation matrix as

$$R = \begin{bmatrix} \cos\theta & \sin\theta \\ -\sin\theta & \cos\theta \end{bmatrix} \qquad \text{(S6.2)}$$

This rotation matrix R is multiplied with the training set χ to provide horizontal/vertical image frame (shown as red points in Figure 1b of the manuscript). The horizontal and vertical lengths of this frame are given as

$A_c = |x_{d2} - x_{d1}|$
$B_c = |y_{d3} - y_{d2}|$

Where x_{d2} and x_{d1} are the x-axis coordinates of the image frame in the horizontal direction, and y_{c3} and y_{c2} are the y-axis coordinates in the vertical direction (see Figure S6.2).

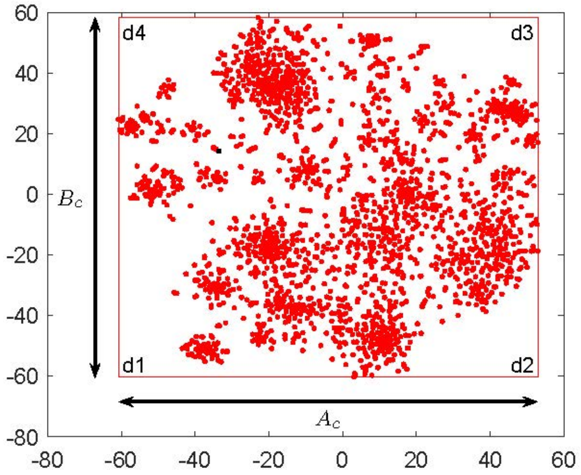

Figure S6.2 Plot showing the rotated data points and the smallest bounding rectangle. The corners of this rotated rectangle are marked as d1, d2, d3, d4. These points are used to compute the width Ac and height Bc.

It is required to convert the Cartesian coordinates to pixel forms for processing. This was done by determining the minimum distance between the two closest points d_{min}. The pixel coordinates can therefore be given as

$$A_p = ceil(A_c \times \frac{Precision}{d_{min}}) \quad \text{(S6.3)}$$

Where $ceil()$ is the ceiling value of the product, and precision will define the resolution. In a similar manner B_p can be found as

$$B_p = ceil(B_c \times \frac{precision}{d_{min}}) \quad \text{(S6.4)}$$

These A_p and B_p values will help to convert Cartesian coordinates to pixel coordinates as

$$x_p = round\left(1 + \frac{(x_c - x_{\min}) \times A_p}{x_{max} - x_{min}}\right) \quad \text{(S6.5)}$$
$$y_p = round\left(1 - \frac{(y_c - y_{\min}) \times B_p}{y_{max} - y_{min}}\right) \quad \text{(S6.6)}$$

Where (x_c, y_c) are x-axis and y-axis coordinates in the Cartesian plane and (x_p, y_p) are coordinates in the pixel frame.
